# Assessing the clinical utility of protein structural analysis in genomic variant classification: experiences from a diagnostic laboratory

**DOI:** 10.1186/s13073-022-01082-2

**Published:** 2022-07-22

**Authors:** Richard C. Caswell, Adam C. Gunning, Martina M. Owens, Sian Ellard, Caroline F. Wright

**Affiliations:** 1 Exeter Genomics Laboratory, Royal Devon University Healthcare NHS Foundation Trust, Exeter, EX2 5DW UK; 2 Institute of Biomedical and Clinical Science, University of Exeter School of Medicine, Exeter, EX2 5DW UK

**Keywords:** Variant classification, Variant interpretation, Missense variant, Pathogenicity, Prediction, Modelling, Protein structure, Genomic medicine

## Abstract

**Background:**

The widespread clinical application of genome-wide sequencing has resulted in many new diagnoses for rare genetic conditions, but testing regularly identifies variants of uncertain significance (VUS). The remarkable rise in the amount of genomic data has been paralleled by a rise in the number of protein structures that are now publicly available, which may have clinical utility for the interpretation of missense and in-frame insertions or deletions.

**Methods:**

Within a UK National Health Service genomic medicine diagnostic laboratory, we investigated the number of VUS over a 5-year period that were evaluated using protein structural analysis and how often this analysis aided variant classification.

**Results:**

We found 99 novel missense and in-frame variants across 67 genes that were initially classified as VUS by our diagnostic laboratory using standard variant classification guidelines and for which further analysis of protein structure was requested. Evidence from protein structural analysis was used in the re-assessment of 64 variants, of which 47 were subsequently reclassified as pathogenic or likely pathogenic and 17 remained as VUS. We identified several case studies where protein structural analysis aided variant interpretation by predicting disease mechanisms that were consistent with the observed phenotypes, including loss-of-function through thermodynamic destabilisation or disruption of ligand binding, and gain-of-function through de-repression or escape from proteasomal degradation.

**Conclusions:**

We have shown that using in silico protein structural analysis can aid classification of VUS and give insights into the mechanisms of pathogenicity. Based on our experience, we propose a generic evidence-based workflow for incorporating protein structural information into diagnostic practice to facilitate variant classification.

**Supplementary Information:**

The online version contains supplementary material available at 10.1186/s13073-022-01082-2.

## Background

In an era of rapidly expanding genomic data, the interpretation and classification of novel missense variants remains a perennial challenge for diagnostic laboratories. Over the years, numerous in silico tools have been developed to assess the impact and pathogenicity of amino acid substitutions and aid variant classification. These tools have developed broadly along two lines [1]: first, analysis of conservation in homologous proteins across species, and second, modelling the impact of mutation on the physicochemical properties of the protein. Although many of these tools perform very well, with high sensitivities and specificities in test datasets, their implementation by diagnostic genomics laboratories has nonetheless been variable and often unsystematic. Furthermore, individual tools can often produce conflicting interpretations [2], contributing to a significant proportion of novel missense variants being classified as variants of uncertain significance (VUS).

The great advances in genomic medicine over the last decade have been paralleled by a concomitant rise in the number of experimentally derived protein structures publicly available in the Research Collaboratory for Structural Bioinformatics (RSCB) Protein Data Bank (PDB) [3] (https://www.rcsb.org). Currently, around 17% of residues in the human proteome are represented in experimentally solved protein structures, although the use of comparative modelling can increase the proportion of residues for which reliable structural analysis can be performed to around 50% [4, 5]. The recently released database of structures predicted by the AlphaFold-2 machine-learning method now provides a predicted structure for 98.5% of residues in the human proteome; however, the confidence with which structure can be predicted is highly variable, and only 58% of residues in the AlphaFold database have a predicted Local Distance Difference Test score (pLDDT) of > 70, the lower limit recommended for use in analysis [6, 7]. As such, while the AlphaFold database represents a valuable resource for structural analysis, it provides a relatively modest increase in the proportion of the human genome which can be modelled with confidence [5, 8, 9].

Where experimental or predicted models of suitable quality are available, numerous studies have demonstrated that in silico protein structural analysis can provide diagnostic utility in genomic medicine, by identifying genetic variants that are likely to be deleterious to protein structure and/or function. Examples of genes studied include *MSH2* [10], *PAH* [11], *MLH1* [12], *LDLR* [13], *GABRA2* [14], *MEN1* [15] and various other genes involved in endocrine disease [16]. Furthermore, in silico thermodynamic predictions based on data from many thousands of protein engineering experiments may offer a generic approach to identifying likely loss-of-function missense variants with high specificity [10, 12, 13, 15]. However, despite this literature, structural analysis remains an underused tool in clinical diagnostic laboratories, in part due to lack of confidence in the validity and utility of the approach. This is a particular problem for diagnostic laboratories that routinely analyse genomes, exomes or other large gene panels, where there may be little or no prior specialist knowledge about the biology of genes in which candidate diagnostic variants are identified.

On the basis of this prior work, we reasoned that protein structural analysis has the potential to be of diagnostic utility for investigating novel missense variants that would otherwise be classified as VUS across a wide range of genes. Here, we report a retrospective dataset of candidate diagnostic variants which were subjected to structural analysis within a UK National Health Service (NHS) genomics laboratory over a 5-year period following implementation of the American College of Medical Genetics and Genomics (ACMG) and Association of Molecular Pathologists guidelines on variant classification [17], and subsequently the UK Association for Clinical Genomic Science (ACGS) Best Practice Guidelines for Variant Classification in Rare Disease [18]. As a result of this work, we present here a generic, evidence-based strategy for incorporating protein structural analysis into a diagnostic workflow for genomic medicine, and describe a number of examples in which analysis at both the structural and sequence level has been key to understanding the impact of novel variants on protein function and the likely mechanism of pathogenicity.

## Methods

### Variant identification and preliminary classification

All variants were identified in patients referred to the Exeter Genomics Laboratory (Royal Devon University Healthcare NHS Foundation Trust) from 2016 to 2020 for genetic testing in a variety of conditions. Informed consent for genetic testing was provided by patients or their parents in accordance with the requirements for clinical diagnostic testing provided through the NHS. All testing was conducted in accordance with the Declaration of Helsinki. Variants were detected by targeted next generation sequencing either of whole exomes or of custom gene panels. For cases referred to the laboratory for panel analysis, a clinical summary was reviewed by a healthcare scientist prior to panel selection to ensure that the referral was appropriate and adhered to any relevant best-practice guidelines. For cases referred to the laboratory for exome sequencing, the case was reviewed by a registered clinical scientist prior to testing to select cases with a likely monogenic disease aetiology. Following testing, all results were discussed with the referring clinician before a report was issued. Probes for in-solution capture and enrichment of target sequences were supplied either by Agilent Technologies (Santa Clara, CA, USA) or Twist Bioscience (South San Francisco, CA, USA) and used according to the manufacturer’s instructions. Sequencing was carried out on an Illumina NextSeq500 or 550 instrument using 150-bp paired end reads (Illumina, San Diego, CA, USA). Variants were called in human genome build GRCh37 and annotated using Alamut Batch software (Interactive Software, Rouen, France). Following evaluation by clinical scientists, variants received a preliminary classification based on the available evidence according to current ACMG/ACGS guidelines. In cases where variants were classified as VUS, we then employed the option of referral for further in-house analysis of protein structure to gain insight into the likely impact of the variant. All variants reported in this manuscript have been deposited in the DECIPHER database [19].

### Protein structure data and analysis

For most of the period covered by this analysis, the availability of structural data was queried via the Universal Protein Knowledgebase (UniProtKB) [20] (https://www.uniprot.org) entry for the protein in question, which provided links either to experimental data for the protein itself in the PDB database [3], or to high quality pre-calculated homology models in the SWISS-MODEL Repository [21] (https://swissmodel.expasy.org/repository). Where no experimental or pre-calculated models were available, protein sequence data was submitted to one or more of the protein modelling servers, SWISS-MODEL [22] (https://swissmodel.expasy.org), Phyre2 [23] (http://www.sbg.bio.ic.ac.uk/phyre2) and I-TASSER [24] (https://zhanggroup.org/I-TASSER), for identification of suitable templates for comparative modelling and subsequent construction of predicted models. For comparative single-templated modelling using the SWISS-MODEL server, models were normally constructed using the template yielding the highest overall quality score (GMQE) over the residue of interest and meeting general requirements for reliable prediction (> 30% sequence identity of target and template over modelled region; length of region modelled > 30 residues). For models generated by multi-templated modelling, only residues predicted with ≥ 90% confidence were used in further analyses. In all cases, protein structures were downloaded as PDB files, and the structural context of the residue(s) affected by the variant investigated in detail using Swiss-PdbViewer [25] and/or PyMOL (PyMOL Molecular Graphics System, Version 2.0, Schrödinger LLC; New York, NY, USA). Specific variants were introduced into PDB files by in silico mutagenesis. Initially, variant sequences were uploaded to the SWISS-MODEL server for re-modelling on the appropriate structure of the native protein. During the course of this work, we also introduced routine use of the FoldX modelling suite [26] for in silico mutagenesis, which provides both a PDB file for the variant protein structure and a quantitative prediction of the impact of the variant on thermodynamic stability of protein structure. In all cases, input PDB files were repaired using the FoldX RepairPDB command prior to mutagenesis. The thermodynamic impact of variants was interpreted according to generally accepted thresholds [27, 28], whereby a change in free energy of the variant structure compared to that of the native or input structure (ΔΔ*G*) > 3 kcal/mol was regarded as severely destabilising, 1–3 kcal/mol as destabilising and < 1 kcal/mol as neutral or benign.

Later in the period covered by this analysis, additional online tools became available that have been designed to integrate and streamline parts of the above workflow. These include PDBe-KB [29] (https://www.ebi.ac.uk/pdbe/pdbe-kb/protein), which provides integrated annotation of experimental structural data for a protein; VarSite [30] (https://www.ebi.ac.uk/thornton-srv/databases/VarSite) and VarMap [31] (https://www.ebi.ac.uk/thornton-srv/databases/VarMap), which provide data on structural conservation and homology-based annotation of residue function; and Missense3D [4] (http://missense3d.bc.ic.ac.uk/missense3d), which performs in silico mutagenesis, either from a user-specified PDB file (which may be either an experimental or predicted structure) or from generation of a comparative model using the Phyre2 server, and then evaluates the effect of missense variants on protein structure by a number of criteria. These tools are now routinely used as part of our structural analysis workflow.

## Results

### Protein structural analysis provides clinical utility for variant classification

From cases referred to the Exeter Genomic Laboratory for genetic diagnosis (~ 28,500 tests conducted during the period covered in this report), we identified a total of 99 unique rare or novel variants (93 missense, three in-frame deletions, two in-frame insertions and one in-frame deletion/insertion variant) for which protein structural analysis was performed. In all cases, these were variants for which there was support for pathogenicity from one or more other strands of evidence (e.g. inheritance status, co-segregation in family members, conservation and variation in healthy and affected populations, clinical fit with gene-specific phenotype, genetic heterogeneity of condition) but which remained as VUS following preliminary classification (Additional file 1). Where structural evidence was used in the final classification, this was applied in all but two cases through the ACMG/ACGS moderate evidence of pathogenicity PM1 criterion, which is applicable when a variant is located within a critical and well-established functional domain [18, 19]; two cases (both variants in *ARSL*) in which evidence was applied under the PP3 criterion remained as VUS. Using 3-dimensional (3D) protein modelling within PM1 enables precise positioning of a variant within the functional region of a protein and may also offer mechanistic insights about how a variant alters domain function. We incorporated the use of FoldX into our routine analysis pathway in February 2017, in order to assess the impact of variants on the thermodynamic stability of protein structure in 80 of the cases listed; this tool was used to analyse 80 variants, and reported ΔΔ*G* values are included in Additional file 1. Of the 19 variants where FoldX was not used, 12 pre-dated our use of this tool, and two were of in-frame indels which cannot be analysed directly by FoldX; in the remaining cases, variants occurred in molecular contexts such as extended, unstructured loops or lipid-facing surfaces of transmembrane helices, where FoldX has limited reliability or predictive value.

Evidence from structural analysis was used in the final classification of 64 variants. Of these, 47 were reclassified as pathogenic or likely pathogenic, with the structural data being applied under PM1 as either moderate (13) or supporting (34) based on professional judgement, while 17 cases remained classified as VUS. No variants were reclassified as benign or likely benign following structural analysis. This was largely due to ascertainment bias in the cohort of variants for which structural analysis was requested, all of which had some existing evidence in support of pathogenicity. However, due to the intrinsic limitations of comparative modelling, whereby an apparent lack of structural damage in silico does not necessarily constitute positive evidence against the possibility of such an effect in vivo, we exercised a conservative approach when applying modelling data to variant classification to minimise misclassification. A summary of evidence from structural analysis of each variant, and whether this was or was not used in the final classification, is included in Additional file 1.

**Fig. 1:**
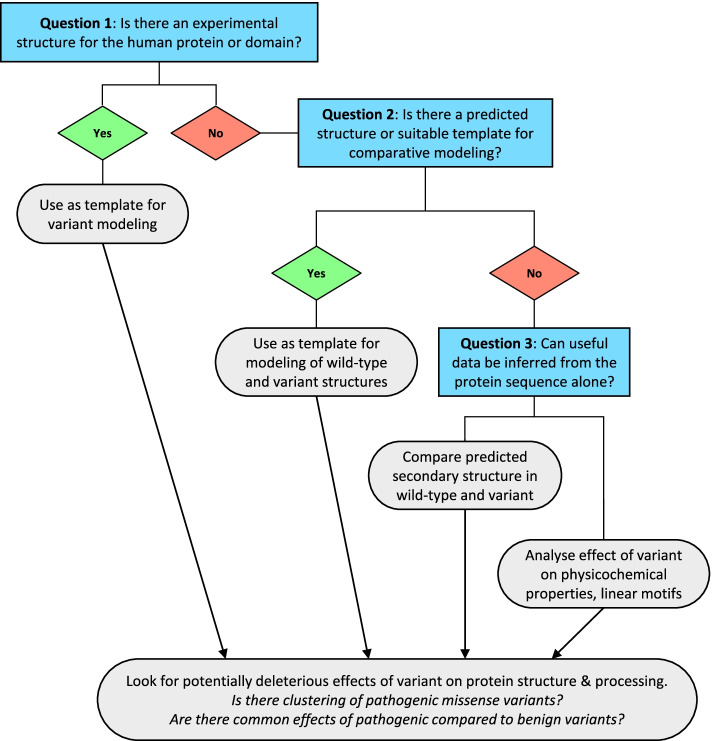
Evidence-based workflow for structural and sequence analysis of missense variants. The generic workflow used for analysis of missense variants proceeds through a short series of questions. Following the initial question (“Is there an experimental structure for the human protein or domain?”), the analysis pathway is then determined by the level of evidence available for each variant, and may differ on a case-by-case basis

Novel variants are by definition unique and uncharacterised, so their analysis is inevitably highly context-specific and can require a high degree of manual intervention and curation. We therefore developed a standardised, evidence-based workflow for the systematic analysis of structural and sequence data as an aid to diagnostic classification of genetic variants (Fig. 1). Although some newer databases and online tools have both expanded the capacity for such analysis as well as streamlining parts of the process, the approach and underlying principles remain the same. The workflow proceeds via a series of yes/no questions according to the level of evidence available for the protein structure and sequence and makes use of a range of freely available software according to the pathway followed. The utility of this approach is demonstrated by four case studies described below.

### Case study 1: Aiding variant interpretation by prediction of loss-of-function through reduced protein stability

Loss-of-function is a common mechanism of disease, but in contrast to protein-truncating variants that result in nonsense-mediated decay, it can be difficult to evaluate whether missense or in-frame deletion/insertion variants will result in impaired protein function. However, thermodynamic destabilisation is a generic mechanism through which missense changes can cause misfolding and/or degradation of a folded protein domain [27, 28], which can be assessed in silico using FoldX or similar tools.

**Fig. 2:**
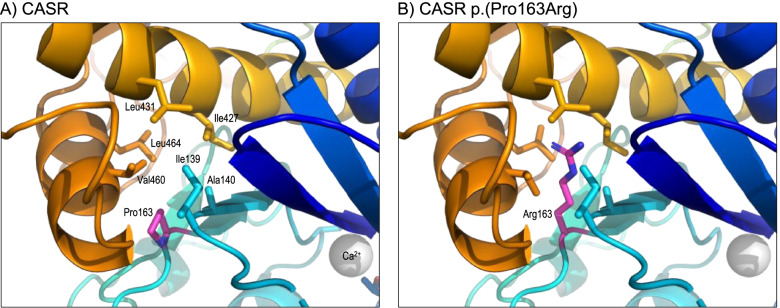
*CASR* NM_000388.3:c.488C > G, p.(Pro163Arg). **A** Structure of the inactive form of CaSR (PDB 5k5t) around Pro163. Protein ribbon is coloured from N-terminal, blue, to C-terminal, red, except for Pro163 (carbon atoms coloured magenta); sidechain atoms of Pro163 and near neighbours are shown in stick format; the grey sphere shows a bound calcium ion. **B** As A, but showing the predicted structure of the p.(Pro163Arg) variant

An example of the value of this analysis was *CASR* variant NM_000388.3:c.488C > G, p.(Pro163Arg), which was identified in a patient referred for genetic testing for Familial Hypocalciuric Hypercalcaemia (FHH). The proband presented with hypercalcaemia and no evidence of parathyroid adenoma or hyperplasia. A calcium infusion test strongly supported a diagnosis of a calcium-sensing receptor disorder rather than primary hyperparathyroidism [32]. The heterozygous p.(Pro163Arg) variant was initially classified as a VUS using the ACMG/ACGS criteria. Subsequent discussions with colleagues in another UK NHS genomics laboratory revealed that this variant had been identified in several individuals and shown to co-segregate with hypercalcaemia in three families. The variant was referred for protein structural analysis to investigate the likely mechanism by which it might alter the domain function. *CASR* encodes the calcium-sensing receptor CaSR, a G protein-coupled transmembrane receptor which senses and regulates the level of extracellular calcium. Inactivating variants in *CASR* cause numerous hypercalcaemic disorders of differing severity, including FHH and neonatal severe hyperparathyroidism, through a loss-of-function mechanism. Conversely, specific activating missense variants cause the opposite phenotype, hypocalcaemia (Bartter syndrome type V) through a gain-of-function mechanism [33]. Experimentally derived protein structures were available for the entire extracellular region in active (calcium bound) and inactive forms (PDB 5k5s and 5k5t respectively) [34]. Inspection of the native structure showed that Pro163 lies in a loop, forming part of a discontinuous calcium binding region in the extracellular region of the protein, with the sidechain buried and surrounded by non-polar amino acids (Fig. 2A). Substitution of the native proline residue by arginine was predicted to result in steric clashes with neighbouring sidechains, while placing the charged, polar sidechain of arginine in the hydrophobic protein core (Fig. 2B). FoldX predicted the ΔΔ*G* values for the variant as ~ 13 kcal/mol in both the active and inactive forms, i.e. an extremely destabilising change [28]. This result was supported by the Missense3D tool, which has previously been shown to have a low false-positive rate for the identification of variants that are deleterious to protein structure [4]. Despite not directly interacting with calcium, the variant was therefore considered highly likely to result in structural destabilisation of the calcium binding region and thus loss-of-function of CaSR, consistent with the hypercalcaemia phenotype. Evidence from the structural analysis was applied as PM1 Moderate under the ACMG/ACGS guidelines, and in combination with co-segregation data allowed the variant to be classified as pathogenic.

### Case study 2: Aiding variant interpretation by prediction of loss-of-function through disrupted ligand binding

**Fig. 3:**
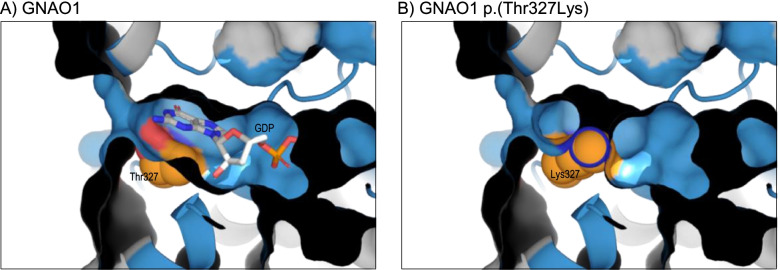
*GNAO1* NM_020988.3:c.980C > A p.(Thr327Lys). **A** Predicted structure of GNAO1 residues 3–347, modelled on template 6crk chain A; the protein is coloured grey by default, with residues of the five nucleotide binding G boxes blue; the view shows both the protein ribbon and surface, sliced through to demonstrate the interior of the binding pocket; the Thr327 sidechain and guanosine diphosphate (GDP) ligand are shown as space-filling spheres, with carbon atoms of Thr327 coloured orange. **B** As A, but showing the predicted structure of the p.(Thr327Lys) variant; note that the novel lysine sidechain is predicted to occlude the binding pocket, with ligand absent from the predicted structure. Models obtained using PDB 3c7k as template were essentially identical to those shown here for 6crk-based modelling

Another potential mechanism by which missense variants can cause loss-of-function is through disruption of ligand binding, which can most easily be determined through evaluation of the protein structure. One example of this mechanism was a novel heterozygous variant, *GNAO1* NM_020988.2:c.980C > A, p.(Thr327Lys), identified in a child referred for exome sequencing with global developmental delay, central axial hypotonia and hypermobility. *GNAO1* encodes a 354-residue member of the Gα family of guanine nucleotide-binding proteins, and Thr327 is annotated as lying in the last of five guanine-nucleotide binding motifs, or G-boxes. Although its precise function is unclear, pathogenic variants in *GNAO1* are known to cause early infantile epileptic encephalopathy type 17 (EIEE17) [35]. Testing of parental samples showed that the variant had arisen de novo but, under ACMG/ACGS guidelines, was initially classified as a VUS and referred for protein structural analysis to investigate the location and likely impact of the variant. None of the existing experimental models for human GNAO1 provided coverage of the entire nucleotide binding domain or included the bound ligand. Therefore, to ensure that these features were included in the analysis, comparative models were built using two different templates: PDB 3c7k, the structure of mouse GNAO1 (97.7% identity to the human orthologue) in complex with regulatory protein RGS16 [36], and PDB 6crk, the structure of human GNAi1 (73.8% identity to human GNAO1), in complex with the Gβ-γ heterodimer [37]. Modelling of the native sequence on both templates revealed that the bound guanine nucleotide interacts directly with Thr327 (Fig. 3A), and that the novel and long lysine sidechain would occlude the ligand binding pocket, thus preventing nucleotide binding (Fig. 3B). The structural data thus provided evidence that the variant was likely to result in loss-of-function and thus fits with the known gene-disease mechanism consistent with the observed phenotype. The result was therefore applied as PM1 Supporting, allowing a final classification of likely pathogenic. It is notable in this case that FoldX predicted no change in stability as a result of the p.(Thr327Lys) variant as FoldX was unable to model the nucleotide ligand, so the larger lysine sidechain appeared to extend into an empty ligand binding pocket.

### Case study 3: Aiding variant interpretation by prediction of gain-of-function through de-repression resulting in activation

In many cases, the benefit of protein structure modelling in variant classification—above and beyond assessing pathogenicity—is to provide evidence supporting a mechanism of pathogenicity for the variant that is consistent with the known mechanism of that disease. Unlike loss-of-function, however, variants that cause gain-of-function are often hard to interpret as their effects on structure are likely to be more variable, potentially more subtle, and may require retention of the active form and structure of a protein. Moreover, there is some evidence that pathogenicity predictors generally perform worse for non-loss-of-function mechanisms [38, 39], so protein structural analysis may provide greater benefits. In the context of proteins which exist in multiple conformations, preferential destabilisation of the inactive form (or stabilisation of the active form) can shift the normal balance between the two forms favouring increased activity.

**Fig. 4:**
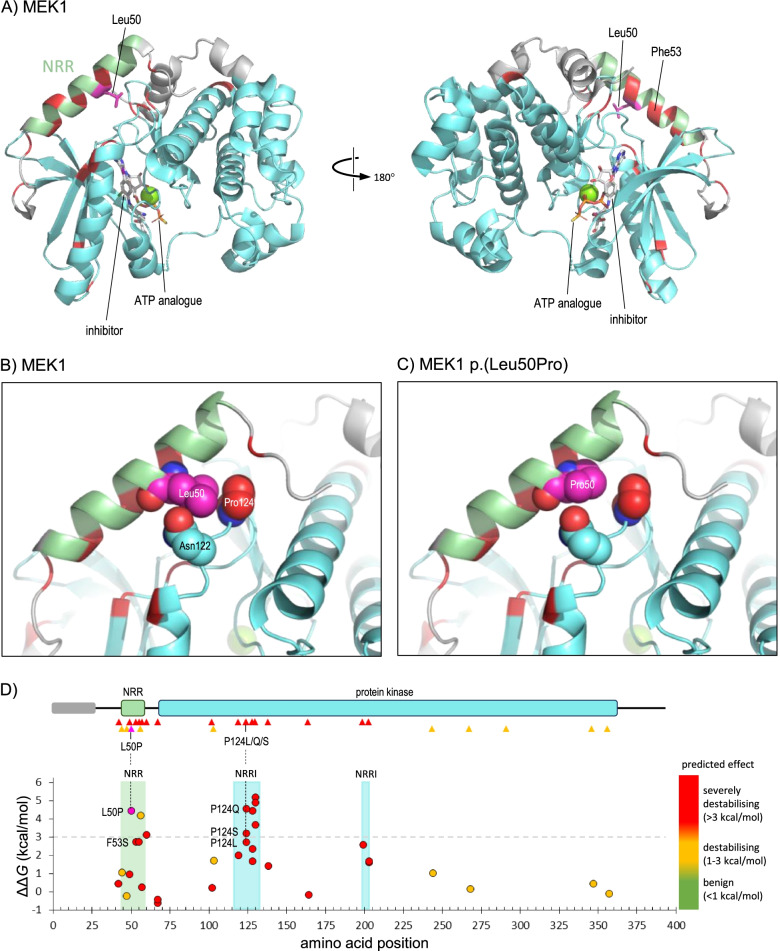
*MAP2K1* NM_002755.4:c.149 T > C p.(Leu50Pro). **A** Structure of MEK1 residues 39–381 in complex with an adenosine triphosphate (ATP) analogue and an inhibitor compound (PDB 3eqc); default colouring is grey, with residues of the NRR (44–58) and kinase domain (68–361) coloured light green or cyan, respectively; additionally, Leu50 is coloured magenta, with the sidechain shown in stick format, while positions of missense variants reported as pathogenic in HGMD (class DM) are coloured red. **B** As A, but magnified to show detail around Leu50, for which all atoms are shown as space-filling spheres; spheres are also shown for sidechains atoms of Asn122 (carbon atoms cyan) and Pro124 (carbon atoms red), which lie in van der Waals contact with Leu50. **C** As B, but showing the predicted structure of the p.(Leu50Pro) variant. **D** The upper part shows the schematic organisation of MEK1; the grey bar indicates a region of predicted disorder (residues 1–27), while green and cyan bars show the NRR and protein kinase domains, respectively; triangles below show the location of variants reported in HGMD (red, pathogenic/class DM; orange, possibly pathogenic/class DM?), while the site of the p.(Leu50Pro) variant is shown by a magenta triangle. The lower part shows the predicted thermodynamic effect of the VUS p.(Leu50Pro) (magenta fill) and all HGMD missense variants (red fill, class DM; orange fill, class DM?) on MEK1 stability calculated in PDB 3uqc, and is aligned to the upper schematic; the light green-shaded region in the graph shows the extent of the NRR (green shading), while cyan-shaded regions show residues of the kinase domain which lie in contact with the NRR (NRRI: NRR-interacting); note that the most destabilising variants, including p.(Leu50Pro), all occur in the NRR or NRRI regions; these include three variants at Pro124, which interacts directly with Leu50 (vertical broken lines)

One example where limited destabilisation can lead to gain-of-function was that of a novel heterozygous missense variant, *MAP2K1* NM_002755.4:c.149 T > C p.(Leu50Pro), which was identified as a de novo variant in an infant presenting with facial dysmorphism (including hypertelorism, down slanted palpebral fissures, prominent forehead and small chin), skin abnormalities, joint hypermobility, macrocephaly and global developmental delay. Variants in *MAP2K1* are known to cause cardiofaciocutaneous syndrome 3 (CFC3; MIM #615,279) [40], in which the above features are typically observed, although our patient had none of the cardiac anomalies which usually occur in this condition. *MAPK1* encodes MAP/ERK kinase 1 (MEK1), which is activated in response to a variety of extracellular signals, and mutagenesis experiments have demonstrated the importance of a short region in the N-terminal of the protein, lying outside the catalytic domain, in maintaining the kinase in an inactive basal state [41, 42]. This region, termed the negative regulatory region (NRR), is the site of one of the previously reported pathogenic variants in *MAP2K1*, namely p.(Phe53Ser) [40]. The crystal structure of MEK1 revealed that the NRR forms an α-helix which lies across the periphery of the kinase domain, locking it in the inactive state prior to activation by tyrosine phosphorylation (Fig. 4A), and leading to the hypothesis that activating variants in the NRR disrupt the inhibitory interaction with the kinase domain [43]. Residue Leu50 lies on the inner surface of the NRR helix, in contact with Asn122 and Pro124 in the kinase domain. Substitution of leucine by proline at this position would be expected to destabilise the helix, while also resulting in loss of contact with Asn122 and Pro124 (Fig. 4B, C); consistent with this, FoldX analysis returned a ΔΔ*G* value of 4.4 kcal/mol, predicting the variant to be severely destabilising to this region of the protein. A value of 2.7 kcal/mol was predicted for the nearby CFC3-associated variant, p.(Phe53Ser) variant, indicating that the p.(Leu50Pro) variant likely has a similar effect in vivo, consistent with disease mechanism and the observed phenotype. Furthermore, an analysis of all variants reported in the Human Gene Mutation Database (HGMD) [44] showed that missense variants that were predicted to cause significant structural destabilisation were confined either to the NRR itself or to residues in the kinase domain which interact with the NRR, consistent with a gain-of-function mechanism whereby pathogenic variants cause local destabilisation within the NRR but not in the core kinase domain (Fig. 4D). The protein structural analysis was applied under PM1 Supporting and the variant was subsequently classified as likely pathogenic.

### Case study 4: Aiding variant interpretation by prediction of gain-of-function through escape from proteasomal degradation

**Fig. 5:**
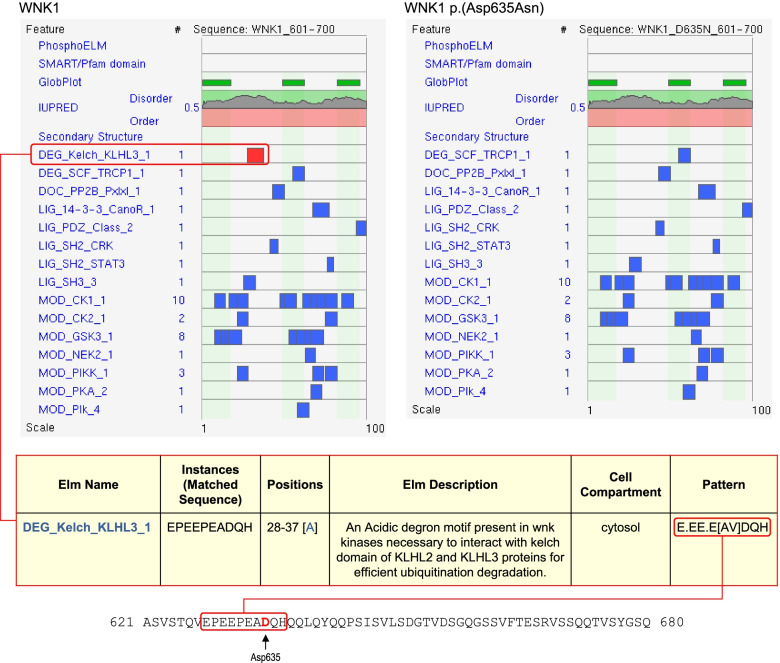
*WNK1* NM_018979.3:c.1903G > A, p.(Asp635Asn). The upper part of the figure shows results of ELM analysis (http://elm.eu.org) for residues 601–700 of native WNK1 (left) and the p.(Asp635Asn) variant (right); upper tracks show predicted sites of phosphorylation (PhosphoELM), conserved domains (SMART/Pfam) and underlying structure or disorder (GlobProt, IUPRED, Secondary Structure tracks); below these tracks are lists of matches to short linear motifs, ranked by score with red shading indicating high confidence; the top hit (and only high confidence scoring motif) in the native sequence was for the Kelch-binding degron motif, DEG_Kelch_KLHL3_1 (boxed in red); this motif was not identified in the variant sequence (upper right panel). The table below shows a detailed description from the ELM server of the DEG_Kelch_KLHL3_1 motif; the right column shows the search pattern for this motif, which is shown below in the context of the WNK1 sequence (Asp635 shown in red font)

A gain-of-function can also occur because of an increased level of active protein, either through increased protein production or reduced protein degradation. An example of this mechanism was that of a novel variant in the *WNK1* gene, NM_018979.3:c.1903G > A, p.(Asp635Asn), which was identified in a patient with a family history of hyperkalaemia and a suspected clinical diagnosis of Gordon’s syndrome. *WNK1* encodes a serine/threonine kinase that plays an important role in the regulation of electrolyte homeostasis, cell signalling, survival and proliferation, and specific missense variants can cause pseudohypoaldosteronism type IIC (PHA2, also known as Gordon’s syndrome), characterised by hypertension and hyperkalaemia, though with normal renal function, and metabolic acidosis [45, 46]. At the time of analysis, HGMD contained only a single *WNK1* missense variant reported in association with PHA2, namely p.(Glu630Lys) [47], and no structure was available for the protein. Furthermore, both Glu630 and Asp635 lie in a region of predicted disorder spanning residues 573–779, for which there were no experimental or high-quality predicted models. Regions of disordered protein may form weak interactions with each other, forming condensates or membraneless micro-organelles that are involved in macromolecular partitioning and other cellular processes [48–51]. Many intrinsically disordered regions also contain short linear motifs (SLiMs) that may act as target sites for protein–protein interactions or post-translational modifications [52, 53]. Importantly, it is becoming increasingly clear that changes to the properties of disordered regions arising from genetic variants play a role in human disease [48, 54–57]. With this information in mind, native and variant WNK1 sequences were scanned using the Eukaryotic Linear Motif resource (ELM) [58] and ScanSite 4.0 [59] to search for potential functional sites within this region. This analysis identified a Kelch-binding degron motif in the native sequence, which was ablated by the p.(Asp635Asn) variant (Fig. 5). The degron is an acidic motif which mediates interaction with Kelch-like protein KLHL3, allowing degron-containing proteins such as WNK1 and the related protein WNK4 to be targeted for proteasomal degradation via KLHL3-Cullin complexes [60]. Variants in the motif thus allow the protein to escape degradation, resulting in accumulation and raised activity of the target protein. Notably, a number of such variants in the degron of *WNK4* (residues 557–566, as annotated by ELM), which cause a related form of PHA2, have been shown to exhibit decreased interaction with KLHL3 [61–63], while the previously-reported p.(Glu630Lys) variant in *WNK1* also affects the degron motif, suggesting a common mechanism of action of these variants. Moreover, around the time of our analysis, a series of pathogenic variants within the degron (or acidic motif) of WNK1 were reported in a cohort of patients with inherited hyperkalaemic hyperchloremic acidosis [64], which included the same p.(Asp635Asn) variant observed in our patient. This report, together with our elucidation of a molecular mechanism for disease, allowed the variant to be classed as likely pathogenic under PM1 of the ACMG/ACGS guidelines, in this case without any 3D protein structural data.

## Discussion

In many cases, a genetic variant can be classified as pathogenic/likely pathogenic, or benign/likely benign using standard automated or semi-automated pipelines or evidence-based algorithms [17, 18] without the need for more in-depth analysis, and indeed, this was the case for the vast majority of variants that were detected in our laboratory during the course of this study. Variants that have either been previously reported to cause disease, or occur in a gene with multiple well-characterised pathogenic variants, or give rise to a very specific and unequivocal phenotype can be relatively straightforward to classify under such procedures due to the weight of existing evidence. However, novel missense variants remain a challenge and when they are of uncertain significance after initial evidence gathering, we have found that there is a strong case for using in silico protein structural analysis as an aid to variant classification applied through the ACMG/ACGS PM1 criterion. While the number of variants reported here is small compared to the total throughput of our laboratory, we have shown that this approach is useful in cases where only a few pathogenic variants have previously been reported in a particular gene, and where missense variants give rise to variable phenotypes or different diseases via alternative mechanisms.

Protein modelling typically requires a substantial level of manual intervention and data curation, and thus, we were prompted to develop a systematic and standardised framework for the incorporation of data from structural analysis into the variant classification pathway (Fig. 1). The proposed pathway uses a number of questions to assess the level and type of evidence available, and while the recent development of new software tools is now helping to automate and streamline this process, the evidence-based principles remain unchanged. Moreover, while in some cases these questions may have a simple yes/no answer, or only limited data availability that suggests an obvious route through the pathway, in many cases there may be a number of possible options for further analysis which should be considered in order to determine the optimal path, as addressed below.

### Question 1: Is there an experimental structure for the human protein or domain?

Our first step in using structural analysis is to determine whether the position of interest is present in an experimental protein structure, as this will normally represent the highest quality of data for further analysis (Fig. 1, box Question 1). Some variant interpretation platforms such as DECIPHER [19] allow users to visualise genetic variants directly in human protein structures where available. For more detailed analysis, the UniProtKB database provides links to experimental structures, along with a summary of the resolution and range of residues covered. More recently, PDBe-KB [29] provides an integrated graphical database of experimental structures of the protein of interest, along with residue-level functional annotation. For detailed analysis, structures of highest resolution (i.e. lowest value in Angstroms) will provide the greatest reliability, although in some cases it may be more informative to use models of lower resolution but that provide more information on the context of the variant. For example, where variants lie at an inter-domain interface, it may be more informative to perform analysis in a structure of lower resolution spanning multiple domains than in a high-resolution structure spanning a single domain. Similarly, if variants are likely to be involved in ligand or protein–protein interactions, structures should be selected that provide information on these interactions. For proteins which exist in multiple conformations, such as enzymes or ion transporters, analysis should be performed in all available conformations, as this may identify conformation-specific effects of variants.

Once identified, protein structures can be downloaded (usually in PDB file format) and analysed in 3D molecular viewers such as PyMOL or Swiss-PdbViewer. Further analysis of the effects of variants on protein structure can be assessed using tools such as FoldX, although it should be noted that FoldX assumes exposed protein surfaces to be in contact with water, and therefore particular caution should be applied when analysing variants within transmembrane regions. Other analysis tools such as DynaMut2 [65], Venus [66] and Missense3D [4] may also be useful in predicting the structural impact of variants, with the latter tool being particularly helpful in a diagnostic setting as it provides information on the structural consequences of a variant without the need for specific expertise in structural biology on the part of the user, as well as a binary classification of the impact of the variant (damaging or not damaging). Moreover, this method has recently been used to evaluate a set of nearly four million missense variants from gnomAD [67], ClinVar [68] and UniProtKB to create the Missense3D-DB [69], thus providing a valuable online resource for understanding the impact of known variants and facilitating the interpretation of novel substitutions. In the case of variants that cause loss-of-function through thermodynamic destabilisation, the effect should be compared to groups of known pathogenic and benign variants over the same region; FoldX is particularly useful in this respect as it allows large numbers of variants to be analysed from input of a single list in text format.

### Question 2: If there is no experimental structure available, is there a suitable template or predicted structure for comparative modelling?

Where experimental structures do not exist, there may be existing predicted models of the human protein which are of sufficient quality for further analysis (Fig. 1, box Question 2). Databases such as the SWISS-MODEL Repository provide high-quality models based on experimental structures of proteins where there is sufficient homology between the query sequence and the template; often, these will be structures of orthologous proteins with close or even complete sequence identity to the human protein that can be used directly as proxies for variant modelling. For example, our modelling of variants in HFN1A and NKX2-1 used structures of mouse or rat orthologues respectively, which were 100% identical to the human proteins over the residues modelled. In some cases, such as that of the GNAO1 p.(Thr327Lys) variant described above, even where there are existing structures of the human protein, it may be preferable to use structures of orthologues or other closely related homologues for comparative modelling if these provide additional context of the impact of the variant. A limitation of these model repositories is that they rely on the prior existence of suitable individual template structures for comparative modelling, whereas the emergence of AlphaFold-2 and other machine-learning methods has enabled the proportion of residues in the proteome for which structures can reliably be predicted to be increased [7, 70, 71]. DECIPHER shows the location of variants in AlphaFold-2 representations and the UniProtKB database provides links to models in the AlphaFold Protein Structure Database (https://alphafold.ebi.ac.uk).

Despite the emergence of the AlphaFold database, there may still be some cases for which there is neither an experimental nor predicted structure covering the residue of interest. As discussed below, this may be due to that fact that the protein is intrinsically unstructured or disordered in the region of interest, in which case meaningful structural prediction will not be possible. In addition, the AlphaFold database currently does not contain models for proteins of > 2700 residues. In such cases, protein sequences can be submitted to modelling servers such as SWISS-MODEL, Phyre2 or I-TASSER for identification of suitable templates, based on sequence similarity and conservation of predicted secondary structure, with subsequent generation of comparative models. Whichever method is used, the quality of modelling is of vital importance in making reliable interpretations of the impact of missense variants. In single-templated models, there should generally be a minimum of 30% identity between query and template sequences (although some structural repeats show a high degree of structural homology with as little as 22–23% sequence identity), with higher levels of identity leading to higher quality of modelling. Alignments between template and query sequence should also be inspected to ensure that the residue of interest is actually modelled directly onto the template sequence, rather than being inserted into the structure as a loop of low confidence prediction. In the case of multi-templated models produced by Phyre2, AlphaFold or other methods, modelled sequences are annotated with a quality or confidence score, and this should always be taken into account when using the models. In the case of Phyre2 models, we normally exclude residues which have been modelled at < 90% confidence, while the recommendation for AlphaFold models is to exclude residues with a pLDDT score < 70. Moreover, in multi-domain proteins, in which individual domains are often joined by flexible linkers, there may be considerable error in how AlphaFold and other methods predict the manner in which these domains interact in 3D space. In AlphaFold models, this uncertainty is expressed in the ‘predicted aligned error’ metric, and should be taken into account particularly when assessing the potential impact of variants which may lie at inter-domain interfaces.

One potential limitation of models predicted by deep-learning methods is that they do not include ligands and typically provide only a single ‘best’ structure for the protein sequence in question. As discussed above, many proteins exist in multiple conformations depending upon ligand binding, post-translational modifications or other processes, and where experimental structures occur it may be informative to perform variant modelling in these different structural forms to fully understand the impact on the protein and its activity. For example, in silico analysis of the hypomorphic ABCC6 variant p.(Arg391Gly) using structures of multiple conformations of the related ABCC1 protein revealed a conformation-specific effect of the variant, thus identifying a molecular basis for a defect in transporter function and explaining the variant’s association with late-onset pseudoxanthoma elasticum [72]. Another current limitation is that deep-learning methods provide only an isolated model of the protein, and therefore lack contextual information on potential protein–protein interactions; however, recent work describing the use of the AlphaFold database in generating models of predicted protein–protein complexes [73, 74] shows promise in this regard.

Following template selection and homology modelling, PDB files can then be analysed in essentially the same way as experimental structures. In the case of FoldX, while this tool has not been formally validated for use on comparative or homology models, it has been shown that such models can provide similar utility to experimental structures in the analysis of variant impact [4], and that FoldX can reliably be used for structural analysis of AlphaFold models over regions of high confidence [8]. Thus the reliability of variant analysis is primarily dependent on the quality of the model itself, rather than on its source.

### Question 3: Can useful data be inferred from the protein sequence alone?

Notwithstanding the ever-expanding resources of experimental and predicted structural data for a protein, there may still be cases where no high-quality models are available over the residue(s) of interest. This may occur either where the protein sequence shares very little homology with proteins or domains of known structure, or where there is in fact no stable underlying structure, i.e. the protein or region is intrinsically disordered. However, this does not necessarily preclude further analysis, as the tendency for a protein sequence to adopt either a stable secondary structure (α-helices, β-sheets, etc.) or to be intrinsically disordered can be predicted with reasonable accuracy from sequence alone. For the former, tools such as PredictProtein [75], PSIPRED [76] and Jpred4 [77] provide predictions of secondary structure and thus can be used to help assess the potential impact of missense variants. With respect to regions of structural disorder, this property can be assessed by online tools such as IUPRED3 [78] or GeneSilico MetaDisorder [79] or by inspection of the MobiDB database [80] entry for the protein. Variants which alter the propensity for disorder have been implicated in the progression of occult macular dystrophy [56] and amyotrophic lateral sclerosis [57]; however, the effects of such variants on protein function will likely be difficult to predict from in silico analysis alone. Conversely, the effects of missense variants on SLiMs and sites of post-translational modification within regions of disorder can be more readily understood, providing utility for tools such as ELM, ScanSite 4.0 and PhosphoSitePlus [81]. In this context, it should be noted that, while in some cases sequence-based analysis might be regarded a last resort for understanding the impact of some variants, in other cases a simple inspection of the UniProtKB entry for the protein may immediately identify a variant as affecting a position of known functional significance, and thus should always be carried out at an early stage of analysis for any variant.

### Limitations to the use of protein structure analysis

Aside from the availability of relevant structures, there are some notable limitations to using a protein structural approach in a clinical diagnostic setting. Although many missense variants will have only a very minor effect on the protein and be insufficiently deleterious to result in a phenotype, some variants that appear in silico to have little or no deleterious effect on the structure of a fully folded mature protein might nevertheless have a major impact on protein folding and/or function in vivo. Variants that lie at the surface of a protein could potentially affect protein–protein interactions, but their impact is likely only to be fully understood either if there is detailed information about the nature of the interaction, or a structure for the relevant protein complex. Searching for such data has recently been greatly facilitated by the development of the PDBe-KB server, which collates data from all known structures of the protein, including sites of ligand and macromolecular interactions, and the VarMap and VarSite databases, which provide functional annotation based on structural homology. Alternatively, surface substitutions may lead to the creation of hydrophobic patches which may result in protein aggregation, such as in the case of the haemoglobin S variant, *HBB* p.(Glu6Val), which causes sickle cell anaemia. A number of bioinformatic tools have been developed to help predict protein aggregation [82], though as yet few if any of these are used routinely in the diagnostic interpretation of missense variants. For these reasons, while a lack of obvious structural damage may be consistent with the presence of a benign variant, the absence of such an effect does not preclude the possibility that the variant may in fact be deleterious. To this end, it has recently been reported that using a combination of sequence conservation analysis and thermodynamic analysis in silico can provide a high degree of discrimination between damaging and benign variants [83], and the development of such methods should help pave the way towards a more integrated use of protein structural data in variant classification.

## Conclusions

We have shown that 3D structural analysis of proteins provides additional clinical utility in the classification of missense variants, particularly in cases of poorly studied genes or those with few known pathogenic variants. We identified numerous missense variants that are predicted to cause complete loss-of-function, either through affecting ligand binding or by severely destabilising the protein; we also identified missense variants that were predicted to cause gain-of-function in critical functional areas of a protein domain. Such evidence can be used directly in variant classification through PM1 in the existing ACMG/ACGS guidelines and is particularly important for determining whether the predicted variant effect is consistent with the known disease-gene mechanism and observed phenotype. In the expectation that this type of analysis will become more widespread, we have outlined some of the most relevant software tools for use in a diagnostic context and presented a generic workflow for implementation of structural analysis in the diagnostic pathway. We hope this work will catalyse the development of a standardised, best practice approach and that wider use of protein structural analysis will ultimately lead to fewer variants remaining classified as VUS.

## Supplementary Information

**Additional file 1.** List of variants subjected to structural analysis, with details of protein modelling, effects observed and contribution of evidence to final variant classification.

## Data Availability

Data required to support the results and conclusions of this manuscript are contained in the manuscript and/or Additional file 1. Individual genetic variants, associated phenotypes and diagnostic classifications have been deposited in the DECIPHER database (https://www.deciphergenomics.org/); database identifiers are shown in Additional file 1. Under the terms of consent for genetic testing, further details of patient cases and sequencing data have not been made publicly available as doing so could potentially compromise the identity and privacy of patients and their families. The following public data resources and tools were used: AlphaFold Protein Structure Database (https://alphafold.ebi.ac.uk) [7] ClinVar (https://www.ncbi.nlm.nih.gov/clinvar/) [69] DECIPHER (https://www.deciphergenomics.org/) [19] DynaMut2 (http://biosig.unimelb.edu.au/dynamut2) [65] FoldX (http://foldxsuite.crg.eu) [26] Eukaryotic Linear Motif resource (ELM) (http://elm.eu.org) [58] GeneSilico MetaDisorder (http://genesilico.pl/metadisorder) [79] The Genome Aggregation database (gnomAD) (https://gnomad.broadinstitute.org/) [68] Human Gene Mutation Database (http://www.hgmd.cf.ac.uk) [44] InterPro (https://www.ebi.ac.uk/interpro). I-TASSER (https://zhanggroup.org/I-TASSER) [24] IUPRED3 (https://iupred.elte.hu) [78] Jpred4 (http://www.compbio.dundee.ac.uk/jpred4) [77] Missense3D (http://missense3d.bc.ic.ac.uk/missense3d) [4] Missense3D-DB (http://missense3d.bc.ic.ac.uk/) [67] MobiDB (https://mobidb.bio.unipd.it) [80] NCBI Conserved Domain Database (https://www.ncbi.nlm.nih.gov/cdd). PDBe-KB (https://www.ebi.ac.uk/pdbe/pdbe-kb/protein) [29] Pfam (http://pfam.xfam.org). PhosphoSitePlus (https://www.phosphosite.org) [81] Phyre2 (http://www.sbg.bio.ic.ac.uk/phyre2) [23] PredictProtein (https://predictprotein.org) [75] PROSITE (https://prosite.expasy.org/prosite.html). PSIPRED (http://bioinf.cs.ucl.ac.uk/psipred) [76] RSCB Protein Data Bank (https://www.rcsb.org) [3] ScanSite 4.0 (https://scansite4.mit.edu) [59] SWISS-MODEL (https://swissmodel.expasy.org) [22] SWISS-MODEL Repository (https://swissmodel.expasy.org/repository) [21] Swiss-PdbViewer (https://spdbv.vital-it.ch) [25] UniProtKB (https://www.uniprot.org) [20] VarMap (https://www.ebi.ac.uk/thornton-srv/databases/VarMap) [31] VarSite (https://www.ebi.ac.uk/thornton-srv/databases/VarSite) [30] Venus (https://venus.cmd.ox.ac.uk/venus) [66]

## Abbreviations

ACGS: Association for Clinical Genomic Science
ACMG: American College of Medical Genetics and Genomics
ATP: Adenosine triphosphate
CFC3: Cardiofaciocutaneous syndrome 3
EIEE17: Infantile epileptic encephalopathy type 17
FHH: Familial hypocalciuric hypercalcaemia
GDP: Guanosine diphosphate
HGMD: Human Gene Mutation Database
NHS: National Health Service of the United Kingdom
PDB: Protein Data Bank
NRR: Negative regulatory region
PHA2: Pseudohypoaldosteronism type IIC
RSCB: Research Collaboratory for Structural Bioinformatics
UniProtKB: Universal Protein Knowledgebase
VUS: Variant(s) of unknown significance
